# Potential association of eEF1A dimethylation at lysine 55 in the basal area of *Helicobacter pylori*-eradicated gastric mucosa with the risk of gastric cancer: a retrospective observational study

**DOI:** 10.1186/s12876-022-02521-5

**Published:** 2022-11-28

**Authors:** Yuka Hirashita, Masahide Fukuda, Masaaki Kodama, Yoshiyuki Tsukamoto, Tadayoshi Okimoto, Kazuhiro Mizukami, Yoshinari Kawahara, Yasuhiro Wada, Sotaro Ozaka, Kazumi Togo, Keisuke Kinoshita, Takafumi Fuchino, Kensuke Fukuda, Kazuhisa Okamoto, Ryo Ogawa, Osamu Matsunari, Koichi Honda, Kazunari Murakami

**Affiliations:** 1 Department of Gastroenterology, Faculty of Medicine, Oita University, 1-1 Hasama-Machi, Oita, 879-5593 Japan; 2 Department of Molecular Pathology, Faculty of Medicine, Oita University, Oita, Japan

**Keywords:** Atrophic gastritis, Gastric cancer, Methylation, Risk factor

## Abstract

**Background:**

Although eradication therapy for chronic *Helicobacter pylori* (*H. pylori*) reduces the risk of gastric cancer (GC), its effectiveness is not complete. Therefore, it is also critically important to identifying those patients who remain at high risk after *H. pylori* eradication therapy. Accumulation of protein methylation is strongly implicated in cancer, and recent study showed that dimethylation of eEF1A lysine 55 (eEF1AK55me2) promotes carcinogenesis in vivo. We aimed to investigate the relationship between eEF1A dimethylation and *H. pylori* status, efficacy of eradication therapy, and GC risk in *H. pylori*-eradicated mucosa, and to reveal the potential downstream molecules of eEF1A dimethylation.

**Methods:**

Records of 115 patients (11 *H. pylori*-negative, 29 *H. pylori*-positive, 75 post-eradication patients) who underwent upper gastrointestinal endoscopy were retrospectively reviewed. The eEF1A dimethyl level was evaluated in each functional cell type of gastric mucosa by immunofluorescent staining. We also investigated the relationship between eEF1AK55me2 downregulation by CRISPR/Cas9 mediated deletion of Mettl13, which is known as a dimethyltransferase of eEF1AK55me2.

**Results:**

The level of eEF1A dimethylation significantly increased in the surface and basal areas of *H. pylori*-positive mucosa compared with the negative mucosa (surface, *p* = 0.0031; basal, *p* = 0.0036, respectively). The eEF1A dimethyl-levels in the surface area were significantly reduced by eradication therapy (*p* = 0.005), but those in the basal area were maintained even after eradication therapy. Multivariate analysis revealed that high dimethylation of eEF1A in the basal area of the mucosa was the independent factor related to GC incidence (odds ratio = 3.6611, 95% confidence interval = 1.0350–12.949, *p* = 0.0441). We also showed the relationship between eEF1A dimethylation and expressions of reprogramming factors, Oct4 and Nanog, by immunohistochemistry and in vitro genome editing experiments.

**Conclusions:**

The results indicated that *H. pylori* infection induced eEF1A dimethylation in gastric mucosa. The accumulation of dimethyl-eEF1A in the basal area of the mucosa might contribute to GC risk via regulation of reprograming factors in *H. pylori* eradicated-gastric mucosa.

**Supplementary Information:**

The online version contains supplementary material available at 10.1186/s12876-022-02521-5.

## Background

*Helicobacter pylori* (*H. pylori)* infection, which causes atrophic gastritis, has been defined as a sequence of histological events that confers an increasing risk of malignant transformation [1, 2]. Indeed, previous basic studies have provided evidence that infection with *H. pylori* carrying specific virulence factors can lead to gastric carcinogenesis [3, 4]. Although, the success rate of eradication therapy has fallen, due to rising resistance to clarithromycin [5], the provision of eradication for *H. pylori* infection by national health insurance was started in February 2013 to reduce the number of new cases of gastric cancer (GC) in Japan.

Reductions in primary and metachronous GC are expected with *H. pylori* eradication, but eradication alone does not completely eliminate GC risk in patients with atrophic gastric mucosa as precancerous lesions [6]. Even after eradication therapy and follow-up diagnosis with barium and endoscopy, there is an urgent need to improve an effective follow-up strategy to identify patients at high risk for GC.

In previous studies, accumulations of methylation in gastric mucosa after *H. pylori* eradication therapy were correlated with GC risk [7]. Most of these reports pointed out only epigenetic biomarkers, such as DNA or histone methylation, in GC risk related to chronic gastritis [8]. However, it was recently revealed that hundreds and likely thousands of proteins are also methylated at lysine, which leads to activation of cellular pathways, such as those for growth signaling and response to damage from chronic inflammation [9]. Eukaryotic elongation factor 1A (eEF1A) is one of the evolutionarily conserved and fundamental non-ribosomal components of the translational machinery [10]. The expression or upregulation of eEF1A has been reported to be associated with cancer development and invasion in ovarian, breast, lung, prostate, hepatic, and pancreatic cancers [11–14]. Additionally, a recent study showed that dimethylation of eEF1Al lysine 55 (eEF1AK55me2) leads to its activation and utilization to increase translational output and promote carcinogenesis in vivo [15].

Although methylation is recognized as a key mechanism in cancer development in atrophic gastric mucosa [16, 17], how lysin methylations are maintained in atrophic gastric mucosa and contribute to GC development is mostly unclear. In this study, we hypothesized that *H. pylori* infection would contributes to potential GC risk through the dimethylation of eEF1A, an active GTPase form, in gastric mucosa.

The aim of this study was thus to clarify the association between the dimethylation of eEF1A, *H. pylori* infection status, and GC incidence in gastric mucosa. Additionally. We investigated how dimethylated eEF1A contributes to GC development after *H. pylori* eradication in gastric mucosa by focusing on the reprogramming factors Oct4 (octamer-binding transcription factor 4) and Nanog.

## Materials and methods

### Study subjects and sampling

We retrospectively investigated the mucosa of the middle portion of the greater curvature of the gastric corpus, approximately 8 cm from the gastric cardia, in patients who underwent upper gastrointestinal endoscopy with biopsy at Oita University Hospital, Japan, between January 2001 and December 2018. Of the 150 enrolled patients, 27 patients with unknow eradication time and 8 patients after gastrectomy were excluded from this study; the remaining 115 patients were included in this study (Additional file 1: Fig. S1). H*. pylori* infection was determined by culture, histology, and rapid urease test. In *H. pylori*-negative patients, we additionally confirmed serum antibody titer (< 3 U/mL) and the lack of endoscopic atrophy and histologic gastritis (Additional file 1: Fig. S1). The success of *H. pylori* eradication therapy was defined as a negative 13C-urease breath test that was confirmed within 6 months after the therapy. Additionally, to exclude *H. pylori*-reinfected patients, we confirmed absence of *H. pylori* by culture, rapid urease test, and histology in eradicated gastric mucosa.

The biopsy site “B2”, which is recommended by the Updated Sydney System, is assumed to be suitable for evaluating the histological status of fundic glands [18]. Specimens were collected endoscopically from the greater curvature of the gastric middle bodies. Biopsy specimens were immediately fixed in 10% neutral buffered formalin for 24 h and embedded in paraffin wax blocks. Fixed samples were sliced into 3-μm-thick sections and stained with hematoxylin and eosin (HE). Degrees of inflammation, activity, atrophy, and intestinal metaplasia were scored according to the Updated Sydney System (0, none; 1, weak; 2, moderate; 3, marked) [18].

### Immunofluorescent staining

Anti-MUC5AC (1:50, Leica Biosystems), H^+^/K^+^ATPase (1:100; Medical & Biological Laboratories), MUC6 (1:200; BioRad), and PG1 (1:200; Abnova) antibodies were used for immunostaining to differentiate between cell types in the fundic gland. We performed immunohistochemistry using anti-dimethyl-eEF1AK55me2 antibody (1:400; Abclonal), Nanog (1:10,000; Cell Signaling Technology), and Oct4 (1:100; Santa Cruz Biotechnology). All biopsy materials were fixed in 10% buffered formalin for 24 h and then embedded in paraffin. After deparaffinization and subsequent rehydration to remove xylene, endogenous peroxidases were inactivated with 3% hydrogen peroxide solution (Wako, Osaka, Japan). Antigen retrieval was performed at pH 6.0. Next, the sections were incubated overnight with monoclonal anti-MUC5AC, MUC6, H^+^/K^+^ATPase, PG1, and dimethyl-eEF1A primary antibodies, followed by incubation with secondary antibodies (1:1000; Alexa Fluor R488 and 594) at room temperature for 2 h. After washing, images were captured with a Keyence BZ-9000 microscope (Keyence, Osaka, Japan).

### Evaluation of dimethyl eEF1A level

Dimethylation levels of eEF1A were evaluated on surface, middle and basal areas of gastric glands, which were identified by morphological appearance or immunostaining with antibodies for specific markers, MUC5AC (surface area), H + /K + ATPase (middle area), and PG1 (basal area), respectively. After immunofluorescence staining with anti-dimethyl-eEF1A antibody, mean fluorescent intensities on these areas were quantified using the regions of interest (ROI) tool in ImageJ software. Multiple ROIs were set for each area based on marker immunofluorescence in one image, and several images at 4 × magnification were used for each patient. Then, the dimethylation level of eEF1A for each area in each patient was expressed as an average of multiple mean fluorescent intensities from the ROIs.

### Cell culture and CRISPR/Cas9

TMK-1, a gastric cell line, was provided by Dr. H. Ito (Tottori Prefectural Kousei Hospital, Tottori, Japan) with permission from an original provider, Dr. A. Ochiai (National Cancer Center, Kashiwa, Japan). Cells were cultured in RPMI1640 (Sigma) with 10% fetal bovine serum. We established Mettl13, which is the physiologic eEF1A lysine 55 dimethyltransferase [15], knockout TMK-1cells using the CRISPR-Cas9 genome editing system and generated stable Mkettl13 knockout TMK-1cells. Briefly, a single guide (sgRNA) targeting Mettl13 (5′-AAGAAAGCTTTCGAGTGGTATGG-3′) was cloned into a Cas9-expressing plasmid (pSpCas9(BB)-2A-Puro(PX450); Addgene plasmid #62,988), and the plasmid was trandfected into TMK-1 cells, which was subsequently cloned after a 2-day treatment with 2 µg/mL puromycine. Knockout of Mettl13 was confirmed by Western blotting.

### Western blotting

Western blotting was performed as described previously [19]. Cells were lysed on ice for 20 min in SDS-modified RIPA buffer containing protease and phosphatase inhibitor cocktails (cOmplete Mini; Roche Diagnostics, Mannheim, Germany) (PhosSTOP EASYpack; Roche Diagnostics), and then centrifuged at 4 °C at 15,000 rpm for 20 min. The resulting cell lysates (20 µg each) were boiled with Laemmli sample buffer and subjected to SDS-PAGE. The samples were transferred to a polyvinylidene difluoride membrane (Merk Millipore, Darmstadt, Germany), which was blocked for 1 h in Block Ace (DS Pharma, Osaka, Japan) at room temperature, then incubated overnight at 4 °C with primary antibodies against METTL13 (1:1000; Bethyl Laboratories), dimethyl-eEF1A (1:1000; ABclonal Technology), eEF1A(1:1000; Abclonal technology), Nanog(1:1000; Abcom), Oct4(1:1000, Abcom), and GAPDH (1:1000, Santa Cruz Biotechnology, Santa Cruz). After washing with 1 × TBS containing 0.1% Tween 20, the membranes were incubated for 1 h at room temperature with appropriate secondary antibodies [19], followed by rewashing. Finally, the signals were detected using an ECL Western blotting analysis system (GE Healthcare, Piscataway, NJ, USA) in accordance with the manufacturer’s instructions.

### Statistical analysis

We investigated the relationship between the clinical factors and dimethyl-eEF1A level of atrophic gastritis mucosa using univariate and multivariate analysis. All variables are expressed as mean ± standard deviation for continuous data (Table 2). Prior to analysis, continuous data were divided into two groups using average values. Levels of eEF1A dimethylation were compared between groups using analysis of variance or analysis of covariance (for age-adjusted data), followed by the Tukey test. Univariate analyses were performed using the Student *t*-test or the Tukey test for continuous variables and chi-squared test for categorical variables. The results of the multivariate logistic regression analysis are expressed as adjusted odds ratios with 95% confidence intervals. A *p*-value < 0.05 was considered to indicate statistical significance. All statistical analyses were performed using with JMP^®^ 11 (SAS Institute Inc, Cary, NC, USA).

## Results

We retrospectively investigated the gastric corpus mucosa from 115 subjects (11 *H. pylori*-negative, 29 *H. pylori*-positive, 75 post-eradication patients). The characteristic of the patients are provided in Table 1. Updated Sydney System scores for inflammation, activity, atrophy, and intestinal metaplasia (IM) are shown in Table 2. The dimethyl level of eEF1A of each gland area identified morphologically or by immunostaining for specific markers (surface, MUC5A; middle, H + /K + ATPase; and basal, PG1) was evaluated (Additional file 2: Fig. S2, Table 2). In the middle area, dimethyl-eEF1A was not detected in MUC6-positive cells but only in H + /K + ATPase-positive cells (Additional file 2: Fig. S2).

**Table 1:** Patient Characteristics (n = 115)

Characteristics	Value
*HP-negative patients(n* = *11)*	
Age (years)	45.36 ± 16.64
Sex(female/male)	3(27%)/8 (73%)
Endoscopic atrophy* (Close/Open)	8 (100%)/0 (0%)
*HP-positive patients (n* = *29)*	
Age (years)	68.76 ± 10.33
Sex (female/male)	7(24%)/22(76%)
History of gastric cancer (−/ +)	16(55%)/13(45%)
Endoscopic atrophy* (Close/Open)	6 (21%)/23(79%)
*Post-HP-eradication patients (n* = *75)*	
Age (years)	69.52 ± 9.24
Sex (female/male)	26 (35%)/49 (65%)
Incidence of gastric cancer (-/ +)	54 (72%)/21 (28%)
*HP* eradication therapy ** (1^st^/2^nd^)	60(80%)/15(20%)
Period after eradication therapy (years)	3.58 ± 3.34
Endoscopic atrophy* (Close/Open)	23 (35%)/52 (65%)

*Kimura-Takemoto classification, **1st line treatment (PPI, [proton pump inhibitor] clarithromycin, and amoxicillin), 2nd line treatment (PPI, metronidazole, and amoxicillin)

*HP Helicobacter pylori*

**Table 2:** Mean Pathological Features and Levels of eEF1A Dimethylation in Gastric Mucosa

*HP*-negative patients (n = 11)	Mean (SD)
Histological features*	
Activity	0
Inflammation	1.00 (0.000)
Atrophy	0
Intestinal metaplasia	0
*eEF1A dimethylation level*	
Surface	16.91 (6.423)
Middle	27.68 (6.010)
Basal	14.03 (4.882)

*HP*-positive patients (n = 29)	Mean (SD)
Histological features*	
Activity	0.79 (0.774)
Inflammation	1.76 (0.786)
Atrophy	0.55 (0.736)
Intestinal metaplasia	0.07 (0.371)
eEF1A dimethylation level**	
Surface	27.95(10.954)
Middle	34.08 (9.658)
Basal	29.31 (6.187)

Post eradicated patients (n = 75)	Mean (SD)
Histological features*	
Activity	0.04 (0.256)
Inflammation	1.20 (0.637)
Atrophy	0.44 (0.793)
Intestinal metaplasia	0.25 (0.660)
eEF1A dimethylation level**	
Surface	22.52 (6.334)
Middle	32.59 (7.437)
Basal	25.26 (9.700)
Intestinal metaplasia	22.01 (8.86)

*Scored using the Updated Sydney System, **Immunofluorescent intensity

### Difference in eEF1A dimethylation of gastric mucosa by H. pylori infection status

**Fig. 1:**
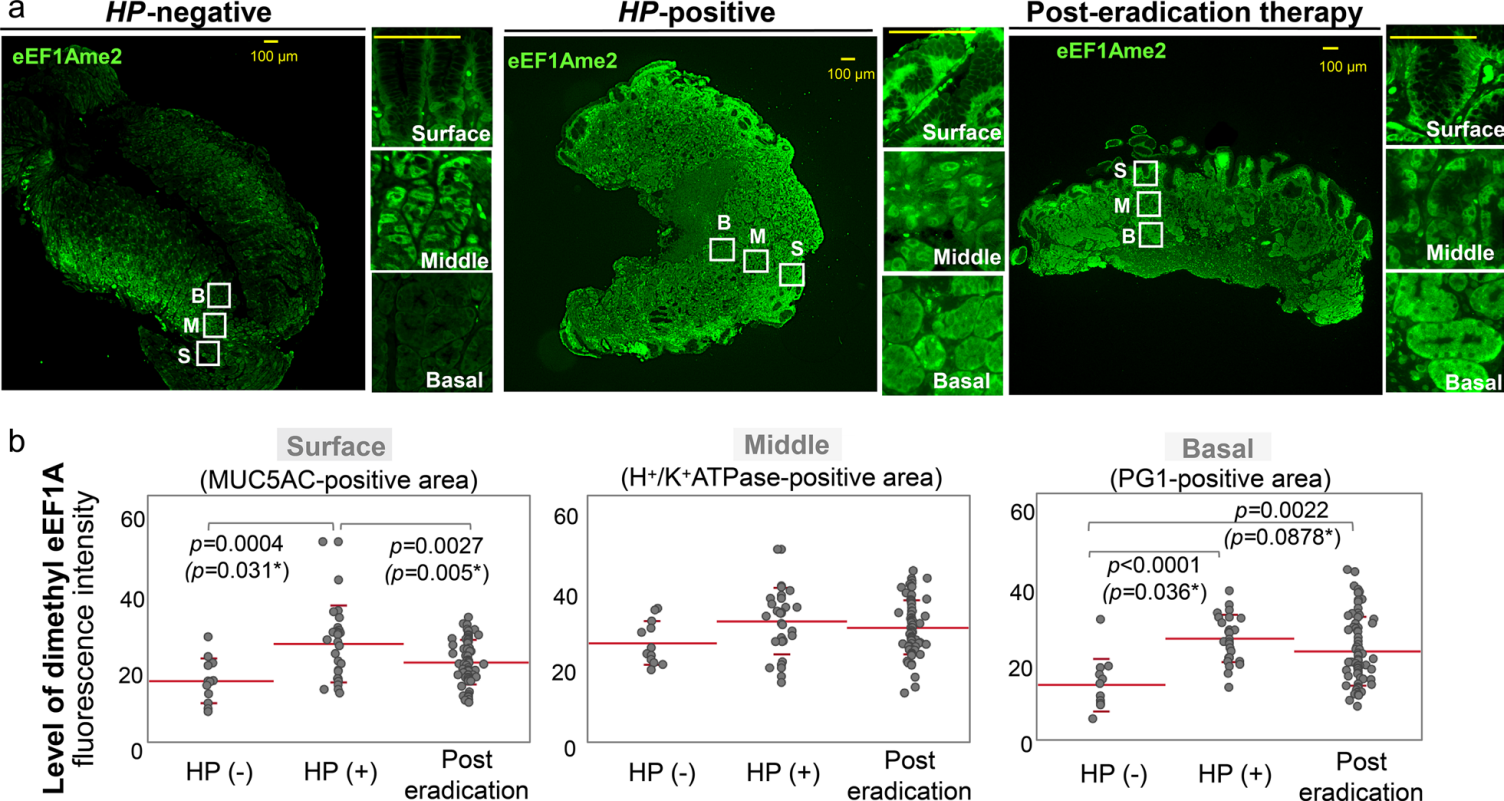
Immunofluorescent staining of dimethyl-eEF1A in *H. pylori*-negative,-positive, and -eradicated gastric mucosa. **A** Representative images of immunofluorescent staining using an antibody against level of dimethyl-eEF1A (eEF1AK55me2). Surface, middle, and basal areas were defined by co-staining with markers specific for each area (see Additional file 1: Fig. S1). **B** Levels of dimethyl-eEF1A in surface, middle, and basal areas were compared between *H. pylori-*negative, -positive, and post-eradicated patients. Differences were calculated by Mann–Whitney U test. *p < 0.05; **p < 0.01; ***p < 0.001

To investigate the association between *H. pylori* infection/eradication and eEF1A dimethylation in gastric mucosa, we first compared the dimethylation levels of eEF1A in each area (MUC5AC-, H^+/^K^+^ATPase-, and PG1-positive cells) among the three groups, *H. pylori*-negative, *H. pylori*- positive, and post-eradicated patients (Fig. 1A). *H. pylori*-positive patients showed higher eEF1A dimethylation than *H. pylori*-negative patients in the surface and basal areas of gastric mucosa (surface: 16.91 ± 6.42 vs. 28.11 ± 10.96, *p* = 0.0004; basal: 14.03 ± 4.88 vs. 29.23 ± 6.33, *p* < 0.0001, respectively). These results suggested that dimethylation of eEF1A is associated with *H. pylori* infection in gastric mucosa.

The dimethylated eEF1A that was caused by *H. pylori* infection was effectively reduced in the surface area (MUC-5AC-positive area) of post-eradicated mucosa (*p* = 0.0027) (Fig. 1B). However, *H. pylori* eradication therapy did not affect eEF1A dimethylation in the basal area (PG-1-positive area) (Fig. 1B). These results suggested that the dimethylation of eEF1A in the basal level remained high in atrophic gastric mucosa even after *H. pylori* eradication therapy.

### Univariate and multivariate analyses of GC and eEF1A dimethylation in basal area of gastric mucosa

**Fig. 2:**
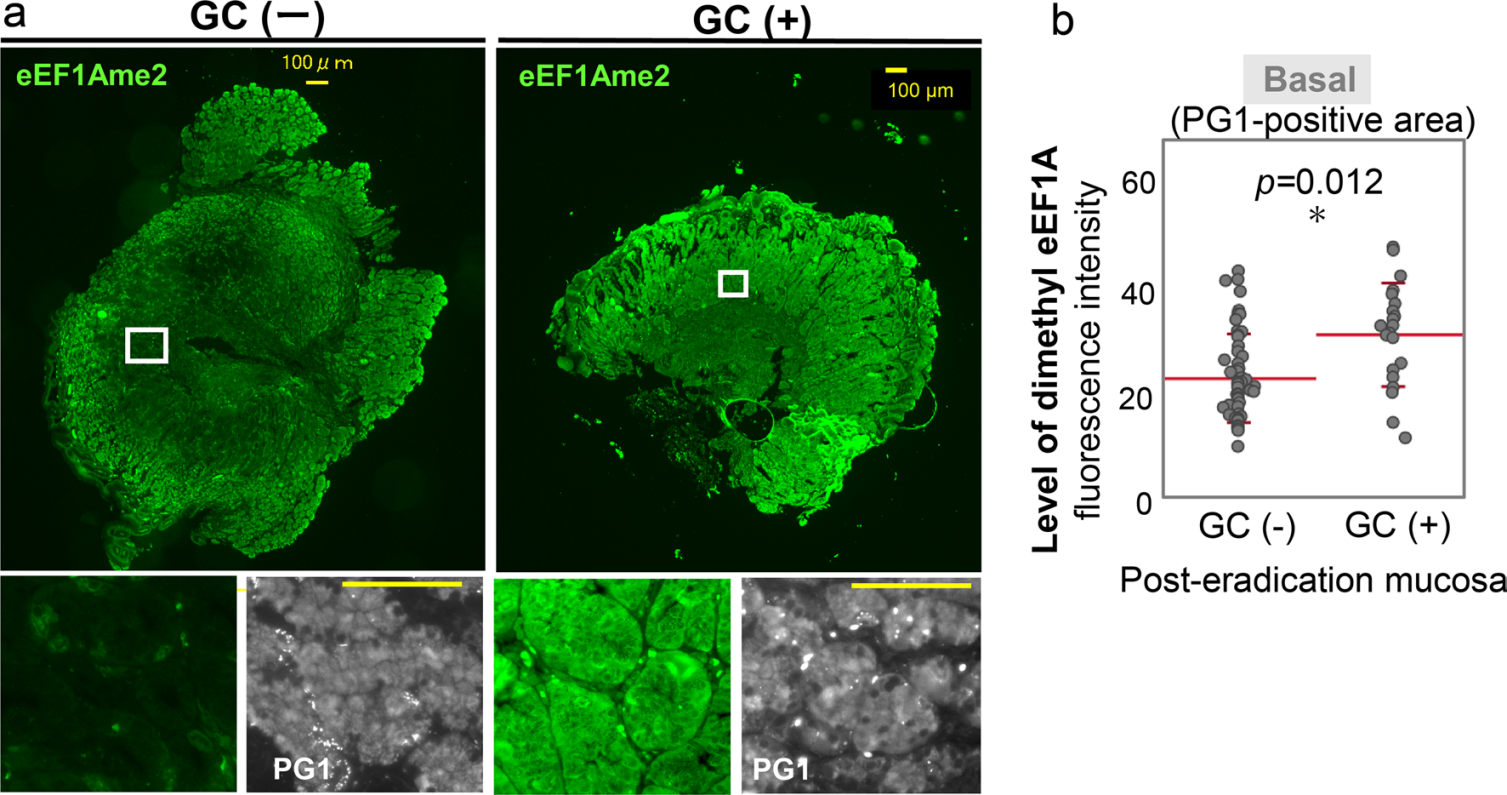
Immunofluorescent staining of dimethyl-eEF1A in *H. pylori*-eradicated mucosa with or without GC incidence. **A** Representative images of immunofluorescent staining using an antibody against level of dimethyl-eEF1A (eEF1AK55me2) and PG-1 for *H. pylori*-eradicated gastric mucosa. **B** Levels of dimethyl-eEF1A in basal areas were compared between post-eradicated patients with or without GC incidence. Differences were calculated by Mann–Whitney U test. *p < 0.05; **p < 0.01; ***p < 0.001

Next, we investigated whether dimethylation of eEF1A in the basal area could cause a risk of cancer in post-eradicated atrophic gastritis mucosa. The influence of known potential risk factors for GC (age, sex, period after eradication therapy, and histological features) and eEF1A dimethyl level in basal mucosa were analyzed in 75 *H. pylori* eradication patients, which included in part those with a history of GC treatment. Univariate analyses showed that severe histological atrophy and high eEF1A dimethylation of the basal mucosal area were the risk factors for GC (Fig. 2, Table 3). Multivariate analysis revealed that not only histological atrophic levels (odds ratio = 9.9493, 95% confidence interval = 1.7096–57.901, *p* = 0.0106) but also high dimethylation of eEF1A in basal mucosal areas (odds ratio = 3.6611, 95% confidence interval = 1.0350–12.949, *p* = 0.0441) were the independent factors related to GC incidence (Table 3).

**Table 3:** Univariate and multivariate analyses of the association between dimethyl eEF1A level and GC

	Univariate			Multivariate	
	GC−	GC +	*P* value	OR (95% CI)	*P* value
Age (years, < 70/ ≥ 70)	26/28	8/13	0.4323	–	–
Sex (female/male)	19/35	7/14	0.8795	–	–
Period after eradication (years, < 3.5/ ≥ 3.5)	32/22	11/10	0.5895	–	–
Histological activity (high/low)	53/1	20/1	0.5054	–	–
Histological inflammation (high/low)	41/13	14/7	0.4219	–	–
Histological atrophy (high/low)	48/6	11/10	0.0009**	9.9493 (1.7096–57.901)	*p* = 0.0106*
Intestinal metaplasia	47/7	17/4	0.5127	–	–
Dimethyl-eEF1A in basal area (low/high)	37/17	6/15	0.0036*	3.6611 (1.0350–12.949)	*p* = 0.0441*

*GC* Gastric cancer, *OR* Odds ratio, *CI* Confidence interval, *p<0.05; **p<0.01

### Accumulation of eEF1A dimethylation and metaplasia in gastric mucosa

**Fig. 3:**
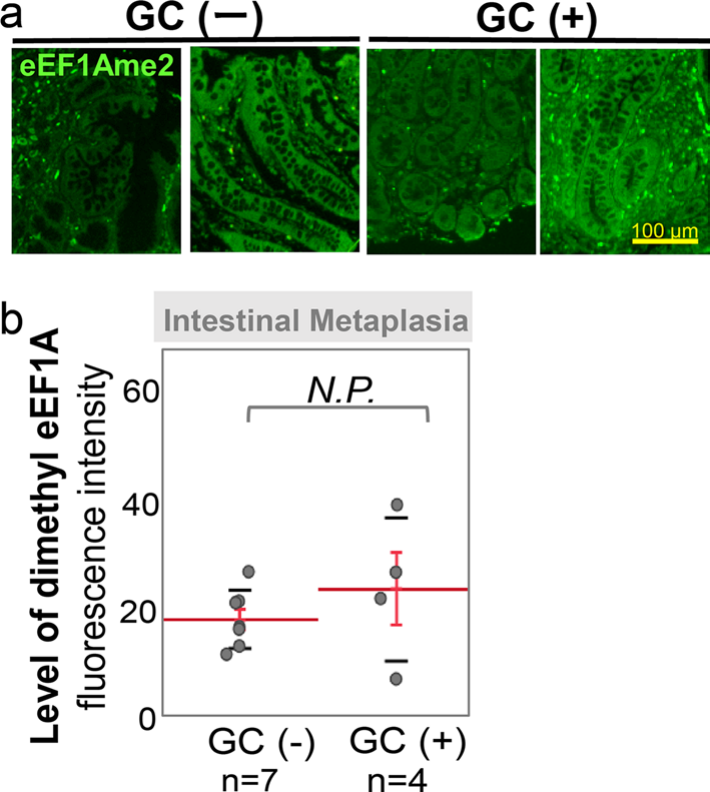
Comparison of dimethyl-eEF1A in area of intestinal metaplasia with or without GC incidence. **A** Representative images of immunofluorescent staining using an antibody against level of dimethyl-eEF1A (eEF1AK55me2) for eradicated gastric mucosa. **B** Levels of dimethyl-eEF1A were compared between patients with or without a incidence of GC using Mann–Whitney U test

Metaplastic lineages have been associated with various injurious scenarios in atrophic gastritis mucosa. We investigated eEF1A dimethylation in the IM area (n = 11) of eradicated mucosa. There was no significant accumulation in eEF1A dimethylation in the IM area compared with other gastric gland cells (mean: 22.01 ± 8.86, Table 2). Although IM is considered an important step in the pathogenesis of GC in atrophic gastric mucosa, there was no significant increase in eEF1A dimethylation in the IM area in eradicated patients with GC incidence (GC(-), n = 7 and GC( +), n = 4) (Fig. 3).

### Association of eEF1A dimethylation with the expressions of reprogramming factors Oct4 and Nanog

**Fig. 4:**
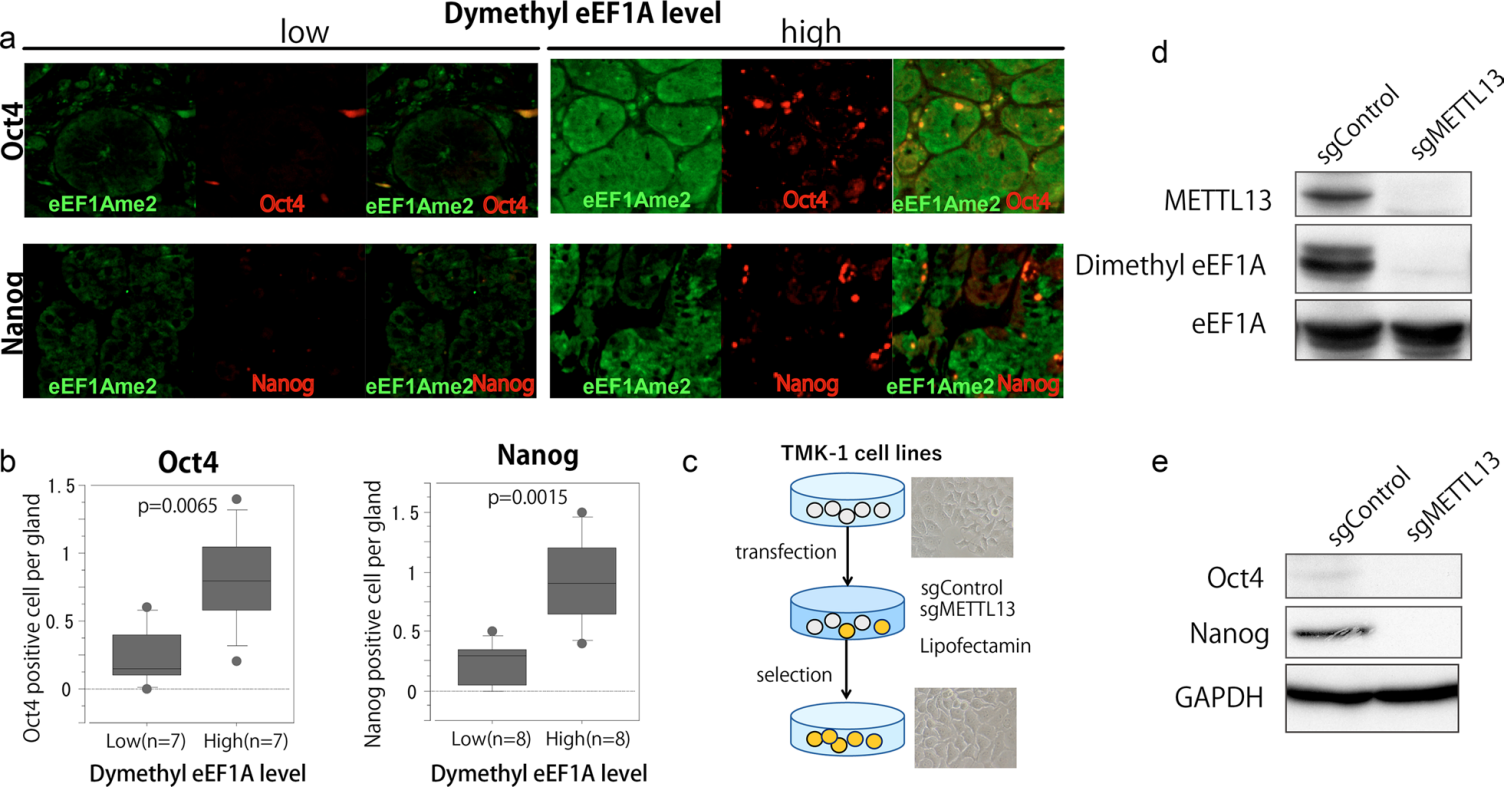
Levels of eEF1A dimethylation were compared with expressions of Oct4 and Nanog in *H. pylori-*eradicated tissues and a Mettl13-deleted cell line. **A** Representative images of immunofluorescent staining using antibodies against dimethyl-eEF1A, Oct4 and Nanog in *H. pylori-*eradicated mucosa. **B** The numbers of Oct4- or Nanog-positive cells per gland in *H. pylori*-eradicated mucosa were compared between patients with low levels (Oct4: n = 7, Nanog: n = 8) and those with high levels (Oct4: n = 7, Nanog: n = 8) of eEF1A dimethylation by Mann–Whitney U test. **C** and **D** Knockout of Mettl13 and downregulation of eEF1A dimethylation in TMK-1 cell were confirmed by Western blotting. **E** Western blotting with antibodies against Oct4, Nanog, and GAPDH revealed that knockout of Mettl13 in TMK-1 cell caused downregulation of Oct4 and Nanog expressions

Next, we investigated whether the expressions of Oct4 and Nanog correlate with dimethylated eEF1A in post-eradicated mucosa. As shown in Fig. 4A, B, the proportion of Oct4- or Nanog-positive cells in eradicated mucosa was significantly higher in patients with high eEF1A dimethylation. However, there was no significant increase in Oct4- or Nanog-positive cells in the IM area (Additional file 3: Fig. S3). To investigate whether dimethylated eEF1A contributes to the expressions of Oct4 and Nanog in gastric cells, we next deleted Mettl13 in a TMK-1 cell using the CRISPR/Cas9 system (Fig. 4C) and analyzed the effect on the expressions of Oct4 and Nanog. As shown in Fig. 4D and Additional file 4: Fig. S4, knockout of Mettl13 led to a decrease of the eEF1A dimethylation level in TMK-1 cells. Interestingly, reduction of dimethylated eEF1A downregulated the expression of Oct4 and Nanog in TMK-1 cells (Fig. 4E). These results suggest that accumulation of dimethylated eEF1A in the basal area of eradicated mucosa is associated with the expression of Oct4 and Nanog, which control reprogramming of various types of differentiated cells, in response to chronic damage induced by *H. pylori* infection.

## Discussion

The presence of *H. pylori* and virulence factors such as CagA, a well-known inducer of chronic inflammation and atrophy, have been reported to be associated with aberrant DNA methylation in gastric mucosa [17, 20, 21]. However, it remains unknown whether *H. pylori* infection also induces aberrant methylation at the protein level, except for histone modification. In this study, we immunohistochemically analyzed the association of *H. pylori* status with the distribution of dimethylated eEF1A, whose function in tumorigenicity has been characterized recently by Liu S et al. [15]. This is first report, to our knowledge, to spatially show the impact of *H. pylori* infection on the level of eEF1A dimethylation in gastric mucosa.

Our results showed that the levels of eEF1A dimethylation in gastric mucosa were different according to the *H. pylori* status (negative, positive, and post-eradication therapy). We found that eEF1A dimethylation of surface and basal cell areas was higher in *H. pylori*-positive patients than in -negative patients, suggesting that *H. pylori* infection induce eEF1A dimethylation of surface and basal cells in gastric mucosa. Indeed, eEF1A dimethylation in *H. pylori-*negative mucosa with adenocarcinoma of the fundic gland type, which is known to be unrelated to *H. pylori* infection, was as low as those in *H. pylori*-negative and cancer-free mucosa (data not shown), supporting our hypothesis that *H. pylori* infection might cause aberrant protein methylation.

Furthermore, not only inflammation but also aging is known to induce accumulation of methylation in multi-organ tissues. Because there were age differences between these three groups (Table 1), we also calculated the age-adjusted levels of eEF1A dimethylation and compared them between these groups using analysis of covariance with the Tukey test. We found the same tendency in both the crude and the age-adjusted models, indicating that age-related differences in methylation did not account for the results (Fig. 1B).

Eradication of *H. pylori* infection has been reported to reduce the risk of GC among asymptomatic patients in high-risk countries [6]. However, long-term studies from Japan revealed that eradication therapy cannot reduce the risk of GC completely [22–24]. In this study, we focused on a subset of atrophic gastritis patients who showed high dimethylation of eEF1A in the basal area of *H. pylori*-eradicated mucosa and found that the high dimethylation of eEF1A in PG1-positive cells, which are chief cells in the basal area of gastric mucosa, correlated with GC incidence in post-eradicated gastric mucosa (Additional file 6: Table S1). Unlike that in the basal area of *H. pylori*-eradicated gastric mucosa, eEF1A dimethylation in the surface area (MUC5AC-positive cells) was strongly associated with the *H. pylori* eradication therapy, i.e., that in the surface area was significantly reduced after eradication regardless of the GC incidence. Considering that the cell lineages in gastric mucosa are known to vary greatly in life span: from 3 to 5 days for surface cells to several months for basal chief cells [25], these different life spans among gastric-cell lineages might cause the specific accumulation of eEF1A dimethylation in the basal area even after eradication therapy. In our results, eEF1A dimethylation in the basal area of eradicated mucosa did not show significant reduction even after the long-term monitoring after *H. pylori* eradication therapy in patients with GC incidence (follow up periods of < 3.5 vs. ≥ 3.5 years, *p* = 0.3908). Although data from further long-term monitoring after eradication therapy is needed, the level of dimethylated eEF1A could be a potential marker for GC risk during the follow up of *H. pylori* eradicated patients. We additionally evaluated the level of dimethylated eEF1A in gastric mucosa using biopsy samples by Western blotting (data not shown). Although dimethyl-eEF1A levels were detectable, they were not appropriate for a cell-by-cell analysis. Further study will be required to use the dimethyl-eEF1A level as a minimally invasive biomarker for the prediction of GC risk in eradicated mucosa.

Although the severity of atrophic damage is already known to be strongly associated with carcinogenesis by inducing various oncogenic alterations, such as overexpression or amplifications, in gastric mucosa [26, 27], the pathogenic mechanism of how *H. pylori* infection induces GC has been a challenging question for decades. Consistent with the previous studies, the present study also showed that the patients with severe atrophic change have GC incidence (Table 3). Additionally, we confirmed that eEF1A dimethylation in the basal area of gastric mucosa is also an independent factor of GC incidence. Previous studies have also shown that damage in the gastric mucosa, including *H. pylori*-induced oxyntic atrophy, initiates mucosal metaplasia and the reprogramming and proliferation of basal cells, and these response are associated with carcinogenesis in atrophic gastric mucosa [28–30]. Although in our results, there was no significant increase in dimethylated eEF1A in the metaplasia area in eradicated gastric mucosa with GC incidence, we showed the association between the expressions of Oct4 and Nanog, which are known as crucial reprogramming factors, and eEF1A dimethylation in the basal area of eradicated gastric mucosa. Additionally, we also showed that dimethyl-eEF1A induced the expression of Oct4 and Nanog in TMK-1 cells. In recent reports, the chief cell has been identified as a cell capable of reprogramming and a metaplastic response to parietal cell damage in atrophic gastric mucosa [28, 31]. Oct4 and Nanog lie in the core of the transcriptional network that controls stem cell pluripotency [32]. Self-renewing progenitor and differentiating cells expressing reprograming factors may be the target for cancer initiation and progression, through the induction of symmetrical cell division [27]. Although their roles in atrophic tissue are largely unknown, these results suggests that high eEF1A dimethylation in the basal area of eradicated mucosa not only could be a useful marker for GC risk, but also might cooperatively contribute to the development of gastric lesions, such as metaplasia and cancer, via regulation of Oct4 and Nanog. Further molecular functional analysis of eEF1A dimethylation, such as METTL13 rescue experiments for METTL13-KO TMK-1 cells, would lead to elucidation of the mechanism of carcinogenesis in *H. pylori* eradicated-gastric mucosa.

The present study has several limitations. First, this was a retrospective observational study performed at a single center. It only included patients who underwent upper gastrointestinal endoscopy for abdominal symptoms or other medical problems reported. Most participants had primary disease; therefore, bias may have occurred. Next, our study contained only 11 patients with IM, 4 of whom had a history of GC treatment (Table 3). In our study, dimethyl-eEF1A was not detected in MUC6-positive cells (Additional file 5: Fig. S5), so we therefore focused only on the fundic gland area. Although there was no significant difference in eEF1A dimethylation in the IM area between patients with or without GC incidence, some IM regions showed a high level of eEF1A dimethylation even after eradication therapy. Because a recent study showed that alterations in methylation were reported to have been observed in regions of IM and to be associated with subsequent dysplasia or GC in gastric mucosa [33], further prospective studies with larger numbers of samples including IM regions in the antrum area of the gastric mucosa may lead us to the discovery of new pathological mechanisms in carcinogenesis in eradicated gastric mucosa.

In conclusion, the results indicated that *H. pylori* infection induces dimethylation of eEF1A in the surface and basal areas of gastric mucosa. Especially, the dimethylation level of eEF1A in the basal area of gastric mucosae may be associated with the expression of reprogramming factors and GC risk in post-*H. pylori*-eradicated mucosa.

## Supplementary Information

**Additional file 1: Fig. S1** Flow chart of patients enrolled in this study

**Additional file 2: Fig. S2** This study profile and immunofluorescent staining of H. pylori-eradicated gastric mucosa. **A** All patients received eradication therapy and were enrolled at least 6 months after establishment of successful H. pylori eradication (n=75). **B** Schematic model of a fundic gland. **C** Representative images of immunofluorescent staining for MUC5AC, MUC6, H+/K+ATPase, PG1, and dimethyl-eEF1A of gastric mucosa are shown. Merging was not detected in the MUC6 and dimethyl-eEF1A cells

**Additional file 3: Fig. S3** Representative images of immunofluorescent staining using antibodies against dimethyl-eEF1A, Oct4, and Nanog in IM area

**Additional file 4: Fig. S4** Image of full-length membranes of Western blotting

**Additional file 5: Fig. S5** Levels of dimethyl-eEF1A in MUC6-positive areas were compared between H. pylori-negative, -positive, and post-eradicated patients. Differences were calculated by Mann–Whitney U test. *p <0.05; **p <0.01; ***p <0.001

**Additional file 6: Table S1**. Raw data of fluorescence intensity of dimethyl-eEF1A

## Data Availability

Not applicable.
