# Supplementary figures and images for: Potential association of eEF1A dimethylation at lysine 55 in the basal area of Helicobacter pylori-eradicated gastric mucosa with the risk of gastric cancer: a retrospective observational study
Source: BMC Gastroenterol. 2022 Nov 28;22:490. doi: 10.1186/s12876-022-02521-5 (PMC9703661; doi:10.1186/s12876-022-02521-5)

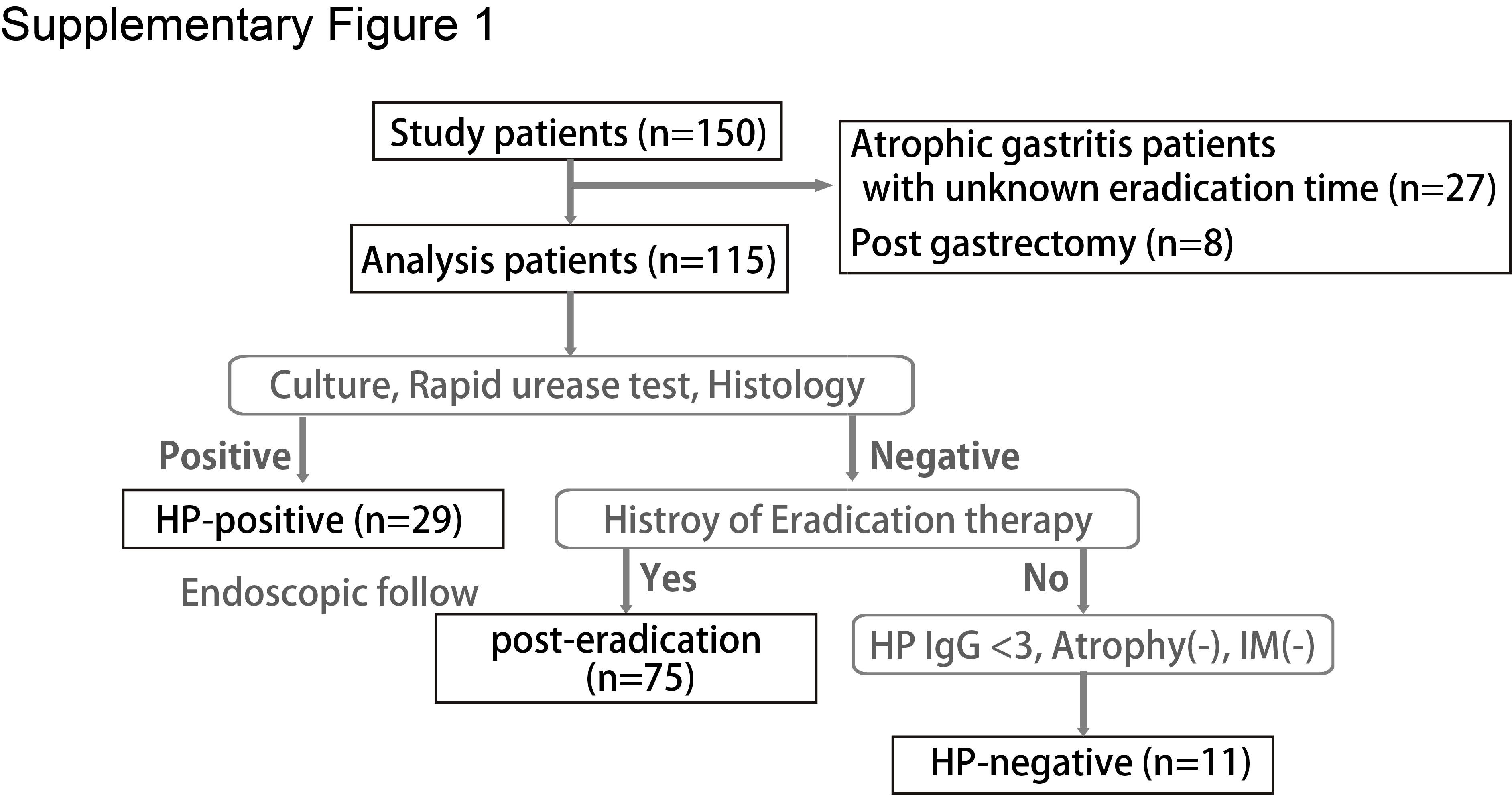

Supplement: Supplementary file 1 — Additional file 1: Fig. S1 Flow chart of patients enrolled in this study [file 12876_2022_2521_MOESM1_ESM.tif]

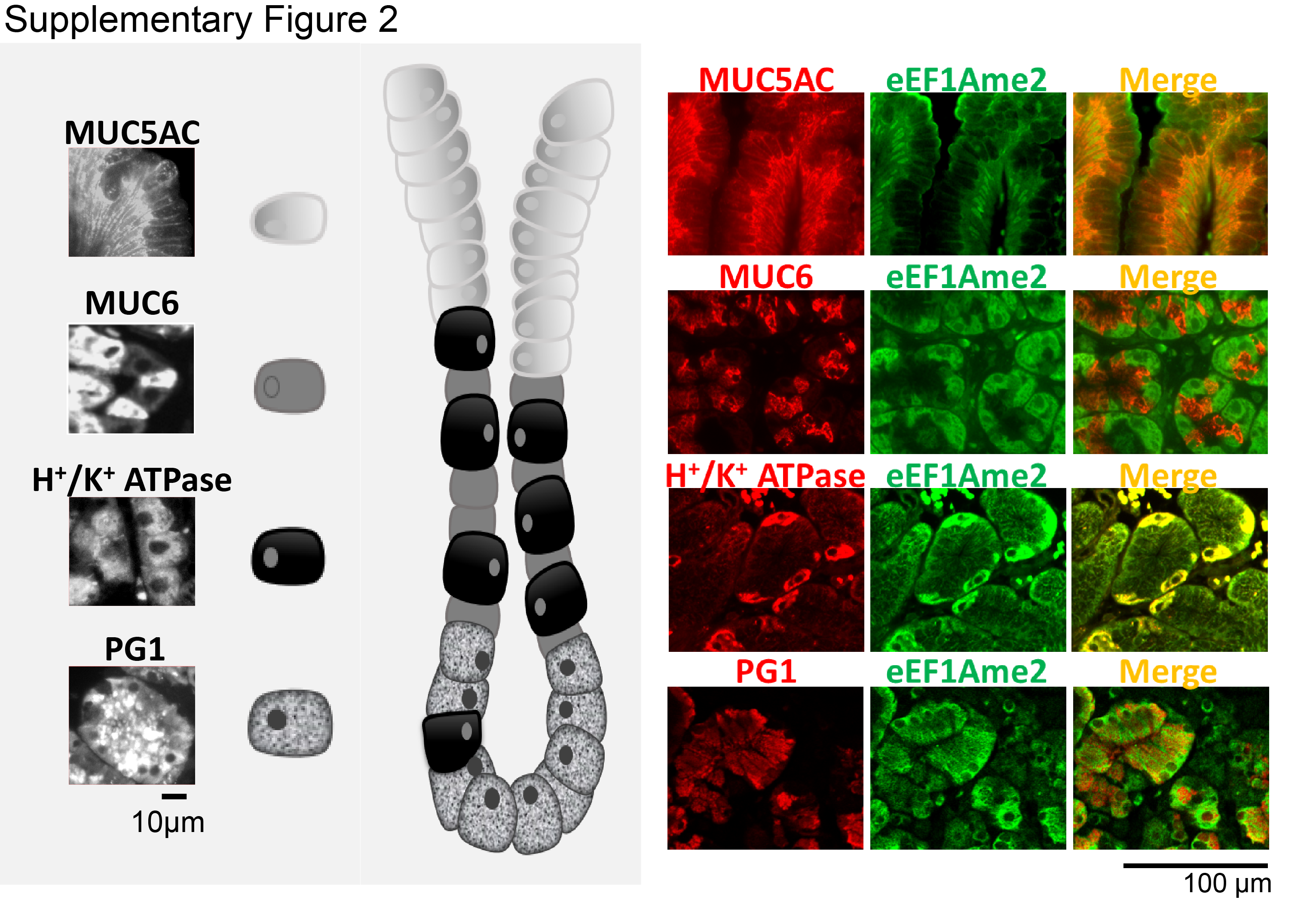

Supplement: Supplementary file 2 — Additional file 2: Fig. S2 This study profile and immunofluorescent staining of H. pylori-eradicated gastric mucosa. A All patients received eradication therapy and were enrolled at least 6 months after establishment of successful H. pylori eradication (n=75). B Schematic model of a fundic gland. C Representative images of immunofluorescent staining for MUC5AC, MUC6, H+/K+ATPase, PG1, and dimethyl-eEF1A of gastric mucosa are shown. Merging was not detected in the MUC6 and dimethyl-eEF1A cells [file 12876_2022_2521_MOESM2_ESM.tif]

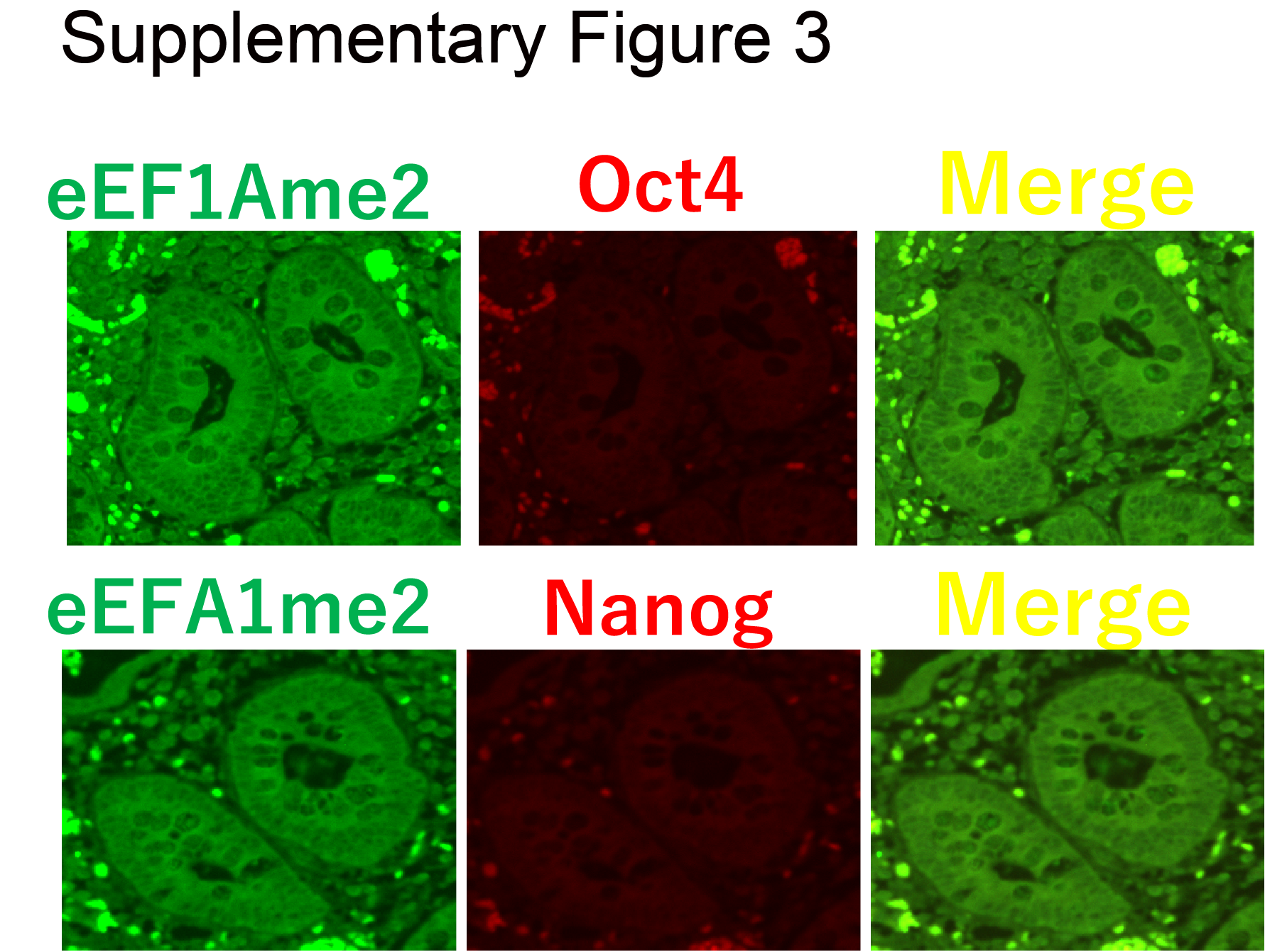

Supplement: Supplementary file 3 — Additional file 3: Fig. S3 Representative images of immunofluorescent staining using antibodies against dimethyl-eEF1A, Oct4, and Nanog in IM area [file 12876_2022_2521_MOESM3_ESM.tif]

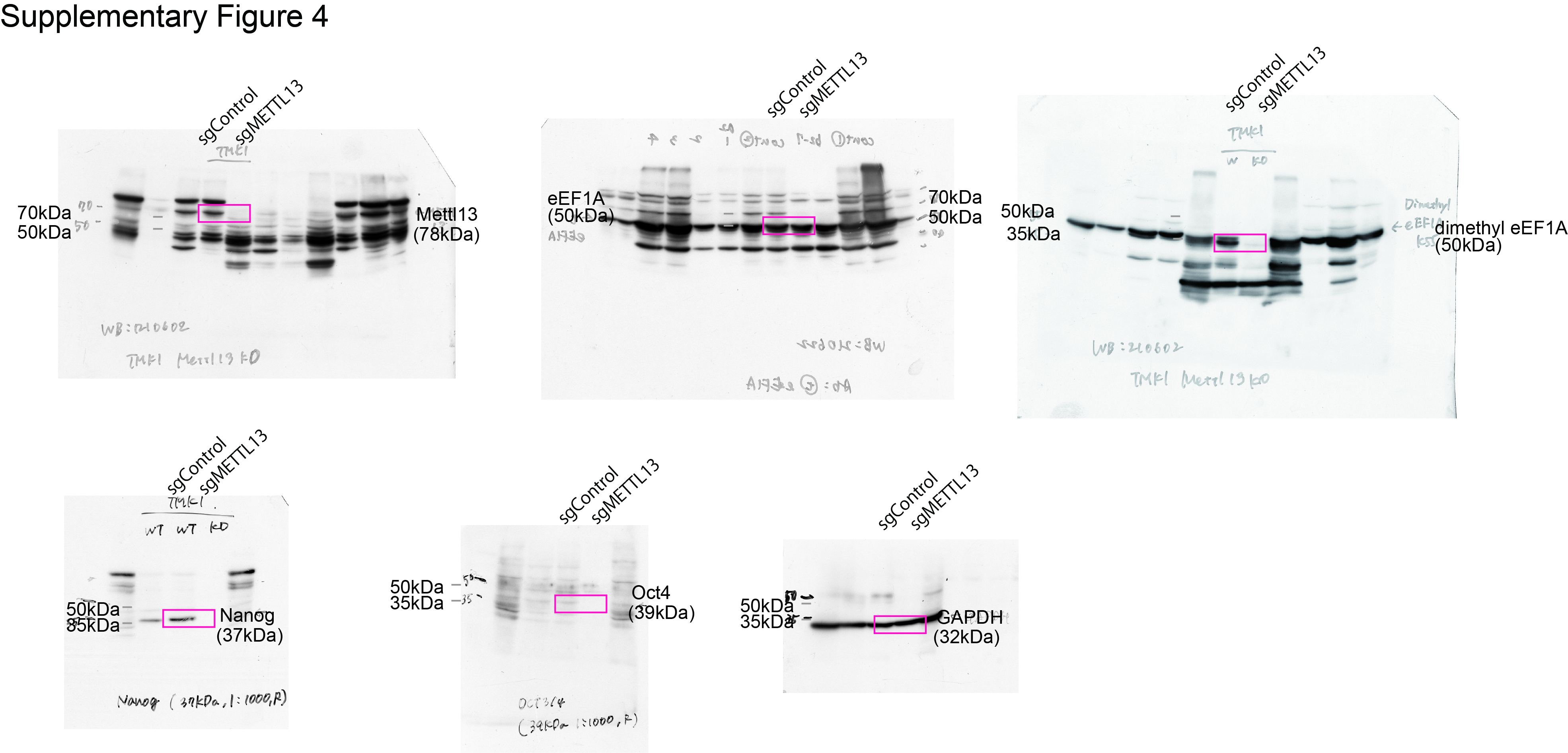

Supplement: Supplementary file 4 — Additional file 4: Fig. S4 Image of full-length membranes of Western blotting [file 12876_2022_2521_MOESM4_ESM.tif]

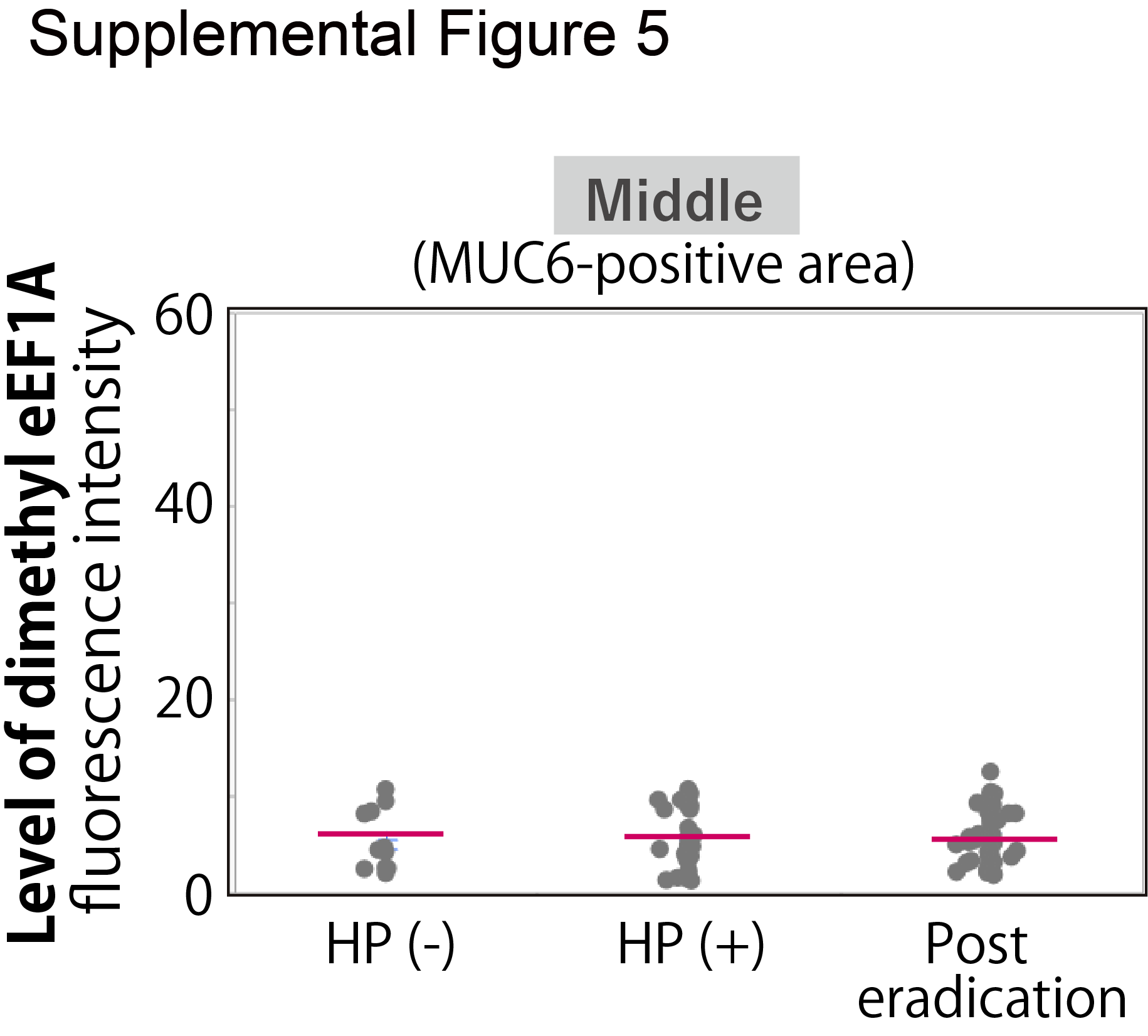

Supplement: Supplementary file 5 — Additional file 5: Fig. S5 Levels of dimethyl-eEF1A in MUC6-positive areas were compared between H. pylori-negative, -positive, and post-eradicated patients. Differences were calculated by Mann–Whitney U test. *p <0.05; **p <0.01; ***p <0.001 [file 12876_2022_2521_MOESM5_ESM.tif]
