# Supplementary material for: Potential association of eEF1A dimethylation at lysine 55 in the basal area of Helicobacter pylori-eradicated gastric mucosa with the risk of gastric cancer: a retrospective observational study
Source: BMC Gastroenterol. 2022 Nov 28;22:490. doi: 10.1186/s12876-022-02521-5 (PMC9703661; doi:10.1186/s12876-022-02521-5)
Supplement: Supplementary file 6 — Additional file 6: Table S1. Raw data of fluorescence intensity of dimethyl-eEF1A [file 12876_2022_2521_MOESM6_ESM.docx]

|  | **eEF1A dimethylation level** | | | |
| --- | --- | --- | --- | --- |
| Sample | Surface  (MUC5AC） | Middle  (H+/K+ATPase) | Basal  (PG1） | MUC6 |
| 1 | 29.34 | 24.67 | 20.47 | 2.05 |
| 2 | 17.20 | 33.21 | 18.09 | 3.26 |
| 3 | 18.86 | 33.92 | 22.52 | 3.53 |
| 4 | 21.94 | 26.15 | 22.88 | 7.58 |
| 5 | 29.44 | 22.25 | 15.87 | 6.35 |
| 6 | 33.06 | 43.78 | 19.75 | 9.96 |
| 7 | 23.14 | 40.33 | 30.70 | 8.94 |
| 8 | 18.93 | 25.05 | 13.61 | 9.93 |
| 9 | 22.97 | 29.72 | 14.20 | 5.32 |
| 10 | 18.25 | 28.86 | 31.22 | 10.22 |
| 11 | 21.66 | 46.37 | 47.72 | 4.28 |
| 12 | 20.37 | 22.58 | 15.71 | 4.18 |
| 13 | 23.05 | 44.99 | 48.32 | 8.73 |
| 14 | 25.52 | 30.67 | 22.05 | 5.91 |
| 15 | 12.01 | 26.28 | 19.67 | 2.19 |
| 16 | 27.32 | 39.46 | 19.62 | 5.07 |
| 17 | 31.75 | 44.66 | 27.05 | 5.56 |
| 18 | 26.23 | 29.05 | 31.97 | 6.06 |
| 19 | 22.77 | 27.13 | 34.83 | 9.50 |
| 20 | 21.57 | 34.32 | 34.19 | 3.88 |
| 21 | 30.12 | 44.15 | 42.70 | 3.70 |
| 22 | 11.83 | 35.25 | 37.33 | 5.87 |
| 23 | 17.86 | 45.72 | 25.67 | 9.24 |
| 24 | 28.70 | 43.95 | 13.58 | 8.29 |
| 25 | 12.66 | 25.33 | 17.59 | 3.44 |
| 26 | 12.70 | 28.30 | 18.79 | 3.91 |
| 27 | 22.30 | 36.12 | 24.67 | 5.88 |
| 28 | 26.12 | 37.12 | 22.10 | 3.29 |
| 29 | 13.83 | 29.66 | 14.91 | 8.26 |
| 30 | 12.99 | 27.17 | 13.00 | 2.10 |
| 31 | 22.69 | 29.04 | 19.09 | 9.25 |
| 32 | 35.11 | 39.89 | 29.09 | 2.10 |
| 33 | 25.54 | 30.00 | 36.00 | 8.11 |
| 34 | 33.31 | 34.63 | 25.72 | 1.78 |
| 35 | 28.20 | 34.22 | 24.44 | 5.69 |
| 36 | 30.22 | 36.67 | 23.00 | 2.02 |
| 37 | 11.96 | 27.78 | 14.61 | 9.48 |
| 38 | 28.98 | 42.25 | 34.30 | 6.93 |
| 39 | 31.30 | 46.29 | 31.59 | 7.44 |
| 40 | 17.18 | 34.21 | 17.28 | 8.34 |
| 41 | 18.36 | 29.66 | 42.03 | 3.73 |
| 42 | 29.12 | 29.46 | 28.03 | 3.02 |
| 43 | 18.20 | 14.14 | 11.21 | 6.95 |
| 44 | 30.23 | 37.13 | 31.21 | 5.18 |
| 45 | 20.94 | 31.71 | 35.88 | 2.77 |
| 46 | 13.48 | 27.94 | 15.04 | 9.50 |
| 47 | 12.97 | 21.94 | 15.00 | 9.50 |
| 48 | 11.20 | 29.91 | 12.64 | 2.76 |
| 49 | 18.16 | 27.35 | 21.67 | 9.93 |
| 50 | 17.98 | 31.30 | 32.99 | 6.01 |
| 51 | 15.74 | 23.06 | 9.53 | 12.47 |
| 52 | 30.15 | 28.02 | 39.19 | 7.15 |
| 53 | 24.92 | 24.94 | 35.33 | 7.44 |
| 54 | 17.58 | 37.15 | 39.69 | 5.90 |
| 55 | 31.65 | 34.46 | 24.00 | 1.78 |
| 56 | 29.94 | 38.13 | 43.69 | 3.04 |
| 57 | 18.92 | 40.21 | 41.78 | 4.94 |
| 58 | 13.00 | 31.21 | 31.84 | 3.11 |
| 59 | 24.23 | 26.32 | 12.89 | 8.01 |
| 60 | 20.55 | 30.91 | 39.90 | 7.23 |
| 61 | 18.98 | 32.47 | 21.14 | 8.16 |
| 62 | 29.13 | 28.13 | 33.09 | 5.11 |
| 63 | 30.13 | 39.12 | 21.21 | 3.10 |
| 64 | 21.98 | 31.89 | 20.04 | 8.16 |
| 65 | 28.88 | 35.00 | 20.94 | 2.11 |
| 66 | 27.82 | 48.48 | 14.81 | 5.37 |
| 67 | 22.03 | 24.95 | 20.22 | 5.00 |
| 68 | 26.43 | 41.67 | 21.06 | 9.24 |
| 69 | 20.22 | 33.61 | 26.37 | 1.98 |
| 70 | 20.03 | 35.70 | 35.25 | 3.63 |
| 71 | 19.57 | 18.57 | 13.42 | 2.77 |
| 72 | 14.16 | 29.61 | 17.11 | 10.34 |
| 73 | 28.70 | 32.51 | 30.91 | 4.34 |
| 74 | 19.98 | 15.77 | 15.36 | 2.35 |
| 75 | 24.32 | 40.50 | 24.15 | 10.20 |
| 76 | 9.44 | 24.67 | 9.93 | 8.36 |
| 77 | 10.99 | 25.15 | 11.11 | 8.12 |
| 78 | 13.60 | 32.35 | 18.57 | 1.97 |
| 79 | 15.98 | 20.65 | 5.99 | 10.63 |
| 80 | 16.96 | 22.62 | 20.94 | 4.61 |
| 81 | 17.19 | 22.14 | 11.14 | 2.48 |
| 82 | 17.33 | 23.54 | 12.50 | 2.40 |
| 83 | 24.05 | 31.15 | 15.97 | 9.43 |
| 84 | 29.61 | 26.84 | 10.38 | 2.56 |
| 85 | 8.62 | 37.44 | 17.26 | 4.39 |
| 86 | 22.26 | 37.90 | 20.51 | 4.19 |
| 87 | 17.11 | 30.22 | 28.57 | 1.40 |
| 88 | 22.08 | 18.79 | 22.26 | 10.20 |
| 89 | 36.66 | 41.54 | 36.82 | 6.66 |
| 90 | 24.89 | 22.08 | 27.87 | 4.89 |
| 91 | 30.70 | 44.21 | 26.13 | 9.33 |
| 92 | 14.86 | 36.55 | 24.30 | 1.50 |
| 93 | 28.17 | 42.89 | 34.53 | 1.25 |
| 94 | 32.22 | 33.66 | 30.37 | 1.98 |
| 95 | 17.86 | 34.95 | 25.32 | 1.50 |
| 96 | 14.86 | 21.17 | 20.85 | 9.55 |
| 97 | 31.41 | 28.57 | 27.75 | 3.96 |
| 98 | 19.93 | 37.95 | 30.67 | 8.94 |
| 99 | 19.37 | 36.50 | 24.32 | 1.50 |
| 100 | 14.86 | 22.18 | 18.85 | 9.55 |
| 101 | 31.41 | 21.01 | 26.75 | 3.96 |
| 102 | 19.93 | 38.20 | 25.17 | 8.94 |
| 103 | 22.82 | 33.29 | 28.51 | 3.33 |
| 104 | 27.09 | 28.74 | 27.23 | 5.91 |
| 105 | 20.21 | 22.89 | 21.43 | 3.70 |
| 106 | 16.11 | 17.08 | 22.24 | 5.68 |
| 107 | 45.59 | 36.82 | 27.65 | 4.46 |
| 108 | 30.62 | 43.87 | 36.03 | 3.27 |
| 109 | 28.79 | 39.11 | 33.23 | 4.72 |
| 110 | 35.02 | 35.70 | 31.18 | 1.19 |
| 111 | 56.33 | 54.47 | 42.32 | 8.93 |
| 112 | 30.89 | 41.50 | 34.80 | 8.56 |
| 113 | 36.97 | 31.53 | 38.33 | 10.65 |
| 114 | 55.56 | 52.33 | 41.66 | 2.20 |
| 115 | 28.17 | 40.70 | 35.02 | 8.56 |

**Supplementary Table**

Raw data of fluorescence intensity of dimethyl-eEF1A
